# Effect of Pentoxifylline Administration on Mast Cell Numbers and Degranulation in a Diabetic and Normoglycemic rat Model Wound Healing

**Published:** 2012-08-30

**Authors:** Saeid Babaei, Mohammad Bayat

**Affiliations:** 1 Cellular and Molecular Biology Research Center, Medical Faculty, Shahid Beheshti University, Tehran, IR Iran

**Keywords:** Pentoxifylline, Mast cells, Diabetes Mellitus, Wound Healing

## Abstract

**Background:**

Wound healing is a complicated and integrated process. Researches in the molecular level on human and animal models have indicated several molecular changes related to the effect of diabetes on wound healing process.

**Objectives:**

Increasing number of researches implicates the influence of mast cells on skin wound repairing. In this study the effect of systemic pentoxifylline (PTX) administration (daily dose of 25mg/kg twice a day, for 7 consecutive days) on normoglycemic (NG) and diabetic (DB) wistar rat’s wound healing by secondary intention was studied.

**Materials and Methods:**

In this study forty eight wistar rats (weighting 250-350g) were divided randomly in two groups: Normoglycemic and diabetic, each group was divided into experimental and control groups, experimental received intraperitoneal (PTX) and controls received distilled water (DW). The number and maturing process of mast cells was evaluated by counting the number of types of mast cells [1][2][3] microscopically and by stereological methods on day 3 and 7 after surgery.

**Results:**

In this study it was cleared that in wound healing process PTX caused increasing the number of type 2 mast cells in all experimental groups(P = 0.00). In normoglycemic experimental group, receiving PTX there was decrease in the number of type3 mast cells, comparing experimental NG groups (P = 0.00).

**Conclusions:**

In all PTX treated groups delay in converting type 2 into type 3 mast cell was seen. Pentoxifylline causes decreasing mast cell degranulation in wound healing process.

## 1. Background

Wound healing is a complicated and integrated process. In all over the world 15% of 200 million diabetic people suffer from diabetic foot disease.[1]. Research in human and animal models has identified a large number of changes associated with diabetes at the molecular level in delayed wound healing.[2] Healing impairment in diabetes is characterized by delayed cellular infiltration, granulation tissue formation, decreased collagen organization, diminished blood flow, increased blood viscosity and, of course, reduced angiogenesis.[3][4][5] Mast cells are found in increased numbers in acute wounds and in certain chronic fibrotic diseases. Growing evidences indicates that mast cells are integrally involved in the process of dermal wound repair. They are resident cells of the normal dermis and have several cytokines stored in their granules that are stimulatory to fibroblasts. They also contain serine proteases that may be involved in remodeling of the extracellular matrix during healing [6]. Mast cells are known to participate in three phases of wound healing: the inflammatory reaction, angiogenesis and extracellular-matrix reabsorption and remodeling. Moreover, there is some evidence that mast cells participate in angiogenesis, stimulation and regulation of endothelial-cell and fibroblast migration and proliferation.[7] Fibrosis can be defined as the replacement of the normal structural elements of the tissue by distorted, nonfunctional and excessive accumulation of scar tissue. Many clinical problems are associated with excessive scar formation. [8] for example keloids, tendon adhesions, scleroderma, liver cirrhosis and hypertrophic scar in the skin. It is also interesting to note that most fibrosis conditions are characterized by an increased number of mast cells.[9][10][11][12] Few studies have shown that pentoxifylline (PTX) therapy improves most problems related to fibrosis conditions e.g., chronic wound healing, venous leg ulcer, tubulointerstitial fibrosis, pulmonary inflammation, DB phlegmona of the foot and sarcoidosis.[13][14][15] PTX belongs to the group of the peripheral vasodilators. It dilates selectively blood vessels of the limbs, brain and retina. The vasodilatative effect of the preparation is a result of inhibition of the enzyme phosphodiesterase and increasing of the concentration of cAMP in the smooth muscle cells of the blood vessel wall. PTX possesses some antiagregant effect also. The latter action is due to the elevation of the cAMP in platelets and (indirectly) to the prostacyclin synthesis potentiation, inhibition of interleukin-1 and 12, inhibition of IL-2 receptors on lymphocytes and TNF-α production on lymphocytes. PTX improves blood rheology by reducing the internal viscosity and mobility of the red blood cell membrane. It improves significantly the microcirculation in the organism.[15][16][17][18] This study was conducted to investigate the effects of PTX administration on mast cells number and degranulation in healing wounds in NG and DB rats.

## 2. Materials and Methods

**Figure 1:**
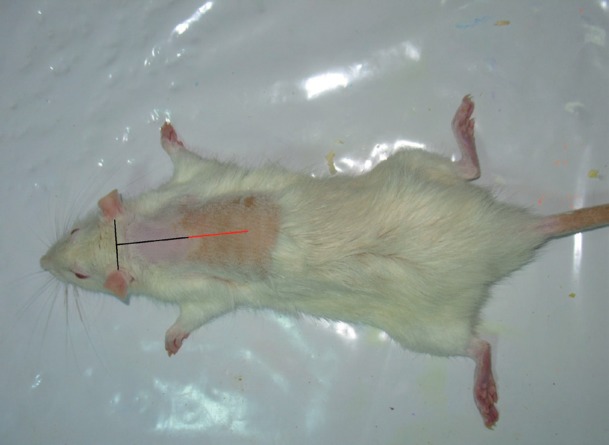
The back of rats was shaved and a linear incision (15mm) was made to the level of the panniculus carnosus muscle. The wounds were not sutured or covered and left to be healed by secondary intention

**Figure 2:**
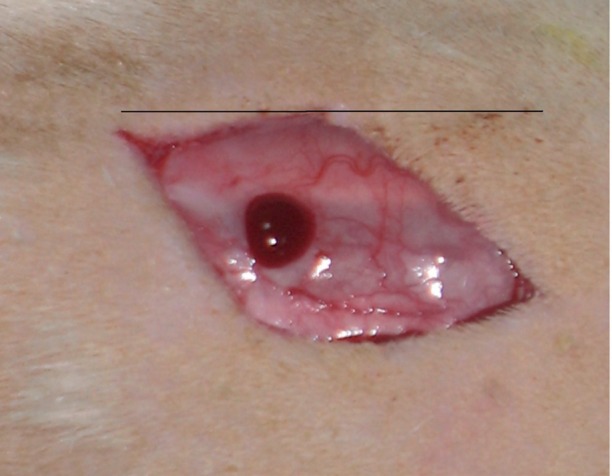
The backs of rats were shaved and a linear incision (15mm) was made to the level of the panniculus carnosus muscle

All procedures in this study were approved by the Institutional Medical Ethics Committee of the University Of Shahid Beheshty (MEC), Tehran, Iran. Forty eight adult male Wistar rats weighing between 250-350g were obtained from Pasteur Institute of Iran and were maintained in our Division of Laboratory where they maintained one per cage with free access to food and water in a room with normal humidity and temperature (22-24 ◦C) on a 12-h light/dark cycle. To provide diabetic group of this study. 24 rats were randomly separated and after a 12-h fasting, were given a single intraperitoneal injection of streptozotocin (Zanosar Pharmacia & Upjohn Co, Kalamazoo, Ml 49001, USA, 55mg/kg body weight solved in distilled water). Seven days after streptozotocin injection, a blood glucose measurement was performed on tail blood using(Supreme Petit, Hypoguard LTD with test strips of Supreme 50 Test code 56)this measurement continued every three days before starting surgery. Diabetes was defined for each animal if blood glucose level was consistently above 300 mg/100ml. During this period DB rats showed clinical signs of diabetes mellitus e.g., polyuria, polyphagia and weight loss. After 30 days of consistent hyperglycemia animal was desired to participate in study. Control groups received distilled water. During the study, blood glucose level of all DB rats was measured every three days. All experimental groups of this study (n = 24) received 25mg/kg PTX, twice a day administered for 7 consecutive days after surgery. Administration of PTX was started 4 days earlier before surgery and continued till the end of the study on day 7. To provide the full-thickness skin linear incision, oriented perpendicular to the long axis of the body each animal was anesthetized with ketamine (50 mg/kg) and xylazine (5 mg/kg). The backs of rats were shaved and a linear incision (15mm) was made to the level of the panniculus carnosus muscle. The wounds were not sutured or covered and left to be healed by secondary intention (Figures 1 & 2).

**Figure 3:**
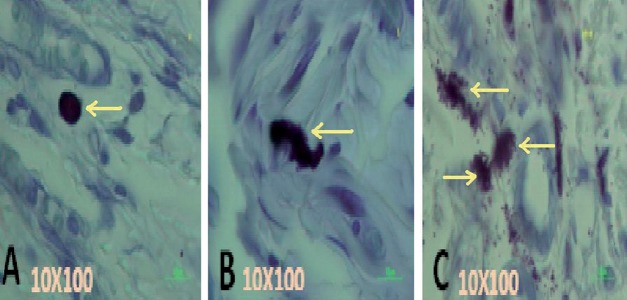
Three Types of Mast Cells in Skin Samples of Normoglycemic Rats Stained With 10% Toluidine Blue and Observed With Magnification10x100 Objective

The wounds were not sutured or covered and left to be healed by secondary intention. Tissues were fixed in formalin (pH = 6.8) and paraffin-embeded. Sections (6µm) were obtained and stained with 10% toluidine blue. According to sensitivity or resistant to formalin, two type of mast cells may be identified [19], in this study, in the rats the formalin resistant cells (connective tissue type) were identified and counted. Considering the degree of granulation in each cell, three types of mast cells were identified in skin samples, representing three stage of mast cell maturation. Intact dark blue-stained cells were considered as type 1 mast cells (Figure 3.A); cells from which some granules had been extruded, but cell outline retained largely intact identified as type 2 mast cell (Figure 3.B); and cells exhibiting extensive and widespread degranulation, with complete or partial disintegration of the cell outline were considered as type 3 mast cells (Figure 3. C).

(A) Type 1 mast cell. (B) type 2 mast cell. (C) Type 3 mast cell To estimate the precise number of all kinds of mast cells in the healing wound area, 3.15mm^2^ area in the border of the incision, near the granulation tissue, or in the area of new collagen formation with x100 objective for each group(n = 6)were photographed and stereological methods were used, these methods may be used to obtain information about three-dimentional structures based on observations made in two-dimentional sections.[20][21][22] In this study 15 double consecutive sections (reference and look up; leaving 5 sections between) leaving 30 sections between double sections, were obtained from each wound and mast cells counting was performed by using specific cell counting grid. Data were evaluated with the SPSS version 16 and all data passed K-S, Leven’s test, Student sample T-Test, ANOVA and One-Way ANOVA, Tukey tests. The criterion for significancy was a P < 0.05. Data are obtained and reported as mean ± standard error of the mean (SEM).

## 3. Results

**Figure 4:**
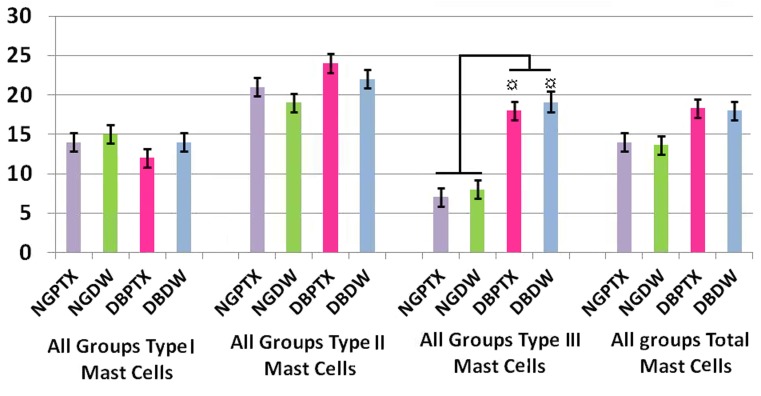
Mean ± SEM for the numbers of types 1, 2 and 3 mast cells, and total number of all types of mast cells in each group on day 3 in 3.15mm2 area of full thickness skin linear incision healing wounds, estimated by stereological methods and magnification x100 objective. The mean number of type 3 mast cells in DB+PTX group is higher than NG+PTX group, (P = 0.000) and NG + DW group (P = 0.000). The mean number of type 3 mast cells in DB+DWgroup is higher than NG+PTX group, (P = 0.000) and NG + DW group (P = 0.000).

**Figure 5:**
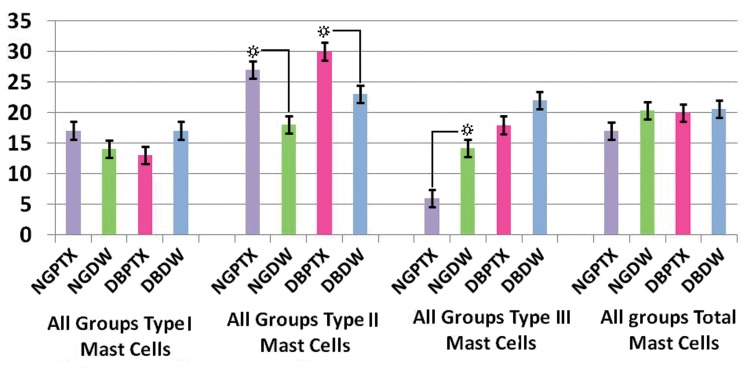
Mean ± SEM for the numbers of types1,2 and 3mast cells, and total number of all types of mast cells on day 7 in 3.15mm2 area of full thickness skin linear incision healing wounds, estimated by stereological methods and magnification x100 objective. The mean number of type 2 mast cells in NG + PTX group is higher than NG+DW group (P = 0.000). The mean number of type 2 mast cells in DB+PTX group is higher than DB+DW group (P = 0.000).The mean number of type 3 mast cells in NG+DW group is higher than NG+PTX group (P = 0.000).

Three of the DB rats died during first week after streptozotocin injection and two of the NG rats were died during anesthesia and postwounding period and were replaced by new ones. Most of the DB and few of the NG rats weighted less at the end of the study compared to the day of surgery, normoglycemics, (P = 0.15) and diabetics (P = 0.07). Blood glucose levels were higher than 300mg/100ml in all DB rats on the day of surgery till the end of the study (day7) and no differences were seen within and between DB groups(P = 0.08). Means ± SD for estimated number of all types of mast cells and the total number of mast cells in 3.15mm^2^ area of each group at days 3 and 7 after linear incision is presented in Figure.4 and Figure 5.

According to statistical analyses on day 7 the mean number of type 2 mast cells in all PTX treated groups(NG + PTX and DB + PTX) is higher than their controls(NG + DW and DB + DW),(P = 0.000). The mean number of type 3 mast cells in NG + DW group is higher than NG + PTX group(P = 0.000).

## 4. Discussion

In present study streptozotocin-induced diabetes is used and this kind of diabetes doesn’t have metabolic or pathologic features of long-term human diabetes, but provides a good model to study acute wound healing events in diabetic rats.[23][24] Considering mere number of counted mast cells and statistical analyses results, it seems that PTX has significantly increased the number of type 2 mast cells in all PTX treated groups (NG + PTX and DB + PTX) on day 7. Counting the mere number of type 3 mast cells and considering statistical analyses results, it seems that although in DB + PTX group reduction of the number of type 3 mast cells comparing control group is happened but this reduction is not significant. However considering the rate of type 3 mast cell decreasing in day 3 and 7 in NG + PTX and DB + PTX groups and comparing them with their controls NG + DW and DB+DW groups (although it is not significant in day3) it seems that PTX has delayed converting type 2 into type 3 mast cells. In NG + PTX group comparing its control group(NG + DW), reduction of type 3 mast cells on day 7 is completely significant and this point may refers that PTX has a distinct reducing effect on the number of type 3 mast cells and in other word in converting type 2 mast cells into type 3 mast cells. Considering all these poins it seems that PTX plays a modulating role in producing type 3 mast cells. A recent study suggested that reducing components of inflammation such as mast cells degranulation may positively affect the wound healing process.[9][25] In general in diabetes mellitus, hyperglycemia by decreasing cell proliferation and affecting collagen synthesis impairs wound healing [3][26][27][28][29][30], in this study, in DB groups, hyperglycemia and increasing the number of type 3 mast cells were the reason for delay in wound healing comparing to NG groups. Furthermore, poor nutrient intake in DB groups partially contributes to wound-repair defect observed in DB rats [2]. Evaluating the process of converting of type 2 into type 3 mast cells in all PTX treated groups, shows that time laps even in DB + PTX treated group will change the converting process as same as the pattern that happened for NG+PTX group. It means that continuing systemic PTX treatment may decreases converting process of type 2 into type 3 mast cells in norm glycemic and diabetic groups.

We found that although PTX decreases the total number of mast cells in all PTX treated groups comparing controls, but this decrease is related to type 1 and may be type 2 mast cells. A recent study reported that mast cells contribute to scar formation during wound healing.[12] Thence when PTX decreases presence of type 3 mast cells during wound healing, it may prevents scar formation by its direct effect on fibroblast proliferation.[31][32] Mast cells contain specific enzymes which are capable of procollagen processing and in this situation abnormal produced peptides are able to stimulate collagen synthesis and lead to fibrosis.[9][10] It has been shown that IL-1α, IL-9, Prostaglandin E2 and monocyte chemotactic protein-1, are different factors that in different ways may enhance mast cell growth by a fibroblast dependant mechanism, mast cell aggregation and granulation.[33] Also an study has shown that PTX may diminish the tissue damage caused by neutrophil inflammatory cytokines by blocking the inflammatory action of IL-1 and TNF-α and as IL-1 enhances mast cells growth, PTX by blocking effect on IL-1, blocks mast cell growth as well.[34] Several studies have reported that In normal human skin wound healing, [34] the rate of monocyte chemo attractant protein-1 is correlated or paralleled with recruitment and degranulation of mast cells.[32][35] Most recent studies have reported that PTX reduces the up regulation of monocyte chemo attractant gene in a dose dependent manner.[36][37] Considering all these reports may leads to the point that PTX plays as a regulator factor to decrease the presence of mast cells in wound healing process by blocking the inflammatory action of IL-1[35] and by reducing the up regulation of monocyte chemo attractant gene in a dose dependent manner.[36][37] In current study it is concluded that PTX has increased the number of type 2 mast cells. More studies are needed to determine other probable mechanisms that PTX may influences in suppressing the presence of mast cells in inflammatory situations.
